# Vascular thoracic outlet syndrome: Registry of 30-years of patient’s outcomes at King Saud University Medical City, Riyadh, Saudi Arabia

**DOI:** 10.15537/smj.2022.43.7.20220336

**Published:** 2022-07

**Authors:** Abdulmajeed Altoijry, Hesham AlGhofili, Kaisor Iqbal, Talal A. Altuwaijri, Sultan Alsheikh, Musaad AlHamzah, Elham Khoujah, Mahmoud T. AbuAlnasr, Badr Aljabri, Mussaad Al-Salman

**Affiliations:** *From the Division of Vascular Surgery (Altoijry, AlGhofili, Iqbal, Altuwaijri, Alsheikh, AlHamzah, Khoujah, Aljabri, Al-Salman), Department of Surgery, College of Medicine, King Saud University, and from College of Medicine (AbuAlnasr), Al-Faisal University, Riyadh, Kingdom of Saudi Arabia.*

**Keywords:** thoracic outlet syndrome, arterial thoracic outlet syndrome, venous thoracic outlet syndrome

## Abstract

**Objectives:**

To outline our experience with both arterial vascular thoracic outlet syndrome (ATOS) and venous TOS (VTOS).

**Methods:**

This was a retrospective review carried out at King Saud University Medical City, Riyadh, Saudi Arabia, from 1992-2022. All patients were diagnosed based on clinical presentation, imaging, and underwent surgical decompression solely via the supraclavicular approach. The median follow-up period was 18 months (range: 4-36 months).

**Results:**

A total of 90 limbs were diagnosed with vascular TOS in 69 patients. Females accounted for 69.6% of the patients and approximately 86.7% had ATOS. All patients were symptomatic and underwent plain thoracic inlet and cervical spine radiography, along with duplex scans in both rest and provocative positions. Total cervical rib resection was carried out in 60% of cases, while 2% had partial resection. First rib resection was carried out in 13.3% of cases and combined cervical and first rib resections were carried out in 23.3%. Vascular procedures were needed for arterial repair in 20% of cases, while venous repair were carried out in 2.2%. No recurrence or post-operative mortality had been reported. Post-operative complications were observed in 18.9% of cases.

**Conclusion:**

Careful patient selection and diagnosis using advanced, but less invasive radiological imaging coupled with adequate surgical treatment can improve the patient’s outcome.

**T**horacic outlet syndrome (TOS) is a combination of symptoms signs that results from compression of the neurovascular bundle as it passes through the thoracic outlet. This condition has been reported in 0.3-8% of the general population.^1^ Clinically, TOS can present as different entities based on the structure that is being compressed. In order of prevalence: compression of the brachial plexus causes neurogenic TOS (NTOS) and makes up 90-95% of cases, compression of the subclavian vein leads to venous TOS (VTOS) and is encountered in 5% of cases, and subclavian artery compression results in arterial TOS (ATOS) and is observed in 1% of cases.^2^ The diagnosis of TOS is challenging because the sensitivity of clinical tests is not ideal, and provocative tests can be positive even in healthy individuals. These factors mandate supplementation by diagnostic imaging to support the diagnosis and to identify potential compression sites.^3^ Because TOS usually affects the young working population, the disabilities that it causes can affect productivity if left untreated.^1^ Adverse outcomes, such as repetitive trauma of the artery wall resulting in stenosis or weakening that leads to post-stenotic aneurysm, can also occur as a result of ATOS; these effects eventually lead to distal embolization and limb-threatening ischemia.^4^ However, NTOS can be treated conservatively with desirable outcomes. Surgical treatment attempts to relieve compression of the neurovascular structures and to reconstruct damaged blood vessels, and can provide fast results.^5^ However, surgical management outcomes of vascular TOS treatment are underreported. A recent meta-analysis included 5 studies on VTOS treatment outcomes, and only 2 studies with a total of 43 patients for surgical treatment of ATOS. We aimed to outline our experience with patients with symptomatic vascular TOS undergoing surgical treatment.

## Methods

This retrospective study was carried out after obtaining approval from the King Saud University Institutional Review Board (project number: E-22-6698) and in accordance with the principles of Helsinki Declaration. All patients provided informed consent.

We included all consecutive patients who underwent surgical decompression of vascular TOS (arterial or venous) between 1992-2022 at King Saud University Medical City, Riyadh, Saudi Arabia. Patients who had pure NTOS were excluded.

Thoracic outlet syndrome was diagnosed based on clinical history, physical examination, and different imaging modalities, such as plain thoracic inlet and cervical spine radiography, duplex scan, computed tomography angiography (CTA), conventional angiography, and venography. Our diagnostic standards were as follows: any compression on vessels in neutral or provocative positions was considered a vascular TOS. Duplex examinations were carried out by an experienced vascular technician using a color Doppler scanner (Philips-ATL HDI-5000, USA) utilizing a linear 4-7 MHz probe. A subclavian artery aneurysm was defined as a dilation of more than 50% the normal size.^6^ We included data such as clinical presentation, investigations, findings, extent of surgical excision, outcomes, and complications. Success was defined as complete relief of vascular symptoms postoperatively, and was confirmed by hemodynamic assessment of the involved limb. We used Derkash’s classification to report patient’s recovery as excellent, good, fair, or poor. Post-operative data were collected during follow-up.

All patients were clinically examined and underwent duplex scanning at one, 6, and 12 months postoperatively, and annually thereafter. The median follow-up period was 18 months (range: 4-36 months). All patients underwent surgery by qualified vascular surgeons. Polytetrafluoroethylene (PTFE) grafts (Gore®, Flagstaff, Arizona, USA) were used during arterial reconstruction. Wallstent-UniTM stents (Boston scientific, Natick, Massachusetts, USA) were used during venous stenting. An English-language electronic literature search of MEDLINE was undertaken to identify prior related studies. The search period was defined by the date of inception of each database. The earliest was for MEDLINE (1945 through Feb 2022). Search terms were thoracic outlet syndrome, thoracic outlet disease, TOS, arterial thoracic artery disease, VTOS, Paget-Schroetter Syndrome, and vascular thoracic outlet syndrome.

### Statistical analysis

Statistical Package for the Social Sciences for macOS (version 28.0; IBM Corp., Armonk, NY, USA) was used to analyze the results. Continuous variables are presented as the means with standard deviations. Categorical variables are presented as percentages.

## Results

A total of 90 cases were diagnosed in 69 patients. A total of 21 (30.4%) patients presented with bilateral symptoms. The median age in our cohort was 32 years (range: 7-70). The majority of patients were female (48/69, 69.6%). Arterial thoracic outlet syndrome was diagnosed in 78 (86.7%) cases. Only 12 (13.3%) cases were VTOS.

Of the 78 ATOS cases, 63 (80.8%) had supraclavicular pulsatile swelling. Upper limb claudication pain was reported in 56 (71.8%) cases. Half of the ATOS cases complained of hand or finger coldness and ischemic color changes. Numbness and paresthesia were reported in 15 (19.2%) cases. Hand muscle atrophy was observed in 11 (14.1%) cases. Critical upper limb ischemia (finger gangrene or rest pain) was found in 10 (12.8%) cases, and these patients had both sensory symptoms and hand muscle wasting. All 12 VTOS cases presented with typical provocative upper limb congestion and swelling consistent with venous compression. Approximately 16.7% VTOS cases presented with acute deep venous thrombosis (DVT).

**Figure 1:**
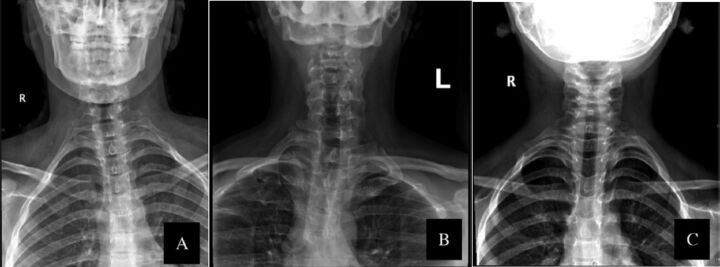
- Plain thoracic inlet films showing: A) bilateral cervical ribs, B) anomalous first rib, and C) unilateral cervical rib.

**Figure 2:**
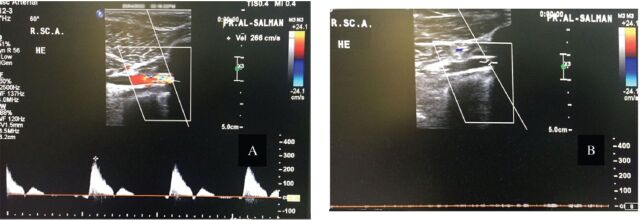
- Duplex scans showing: A) flow in right subclavian artery during rest, B) duplex scan showing right subclavian artery with absent flow during provocative maneuver.

**Figure 3:**
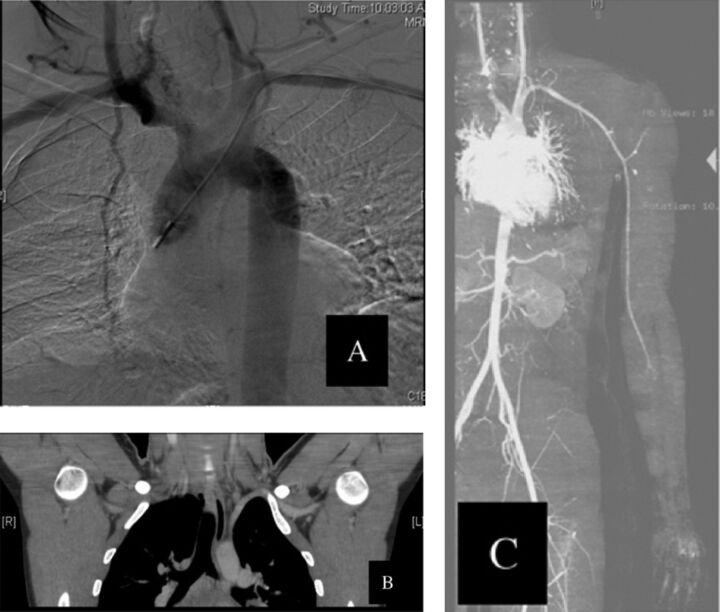
- Conventional angiogram image showing: A) a compressed subclavian artery with post-stenotic dilatation, B) a compressed subclavian artery with post-stenotic dilatation, and C) cessation of blood flow distal to brachial artery.

All patients suspected of vascular TOS underwent both plain thoracic inlet and cervical spine radiography, and duplex scan, as part of the investigation protocol. Plain radiography is helpful as it provides useful information for identifying bony abnormalities in rest and stressed upper limb positions (Figure 1). Duplex scanning successfully identified all ATOS and VTOS cases. Furthermore, it helped detect thrombosis, dilation, or aneurysm (Figure 2). Conventional angiography was carried out until 2006 before we switched to CTA as a second-line confirmatory imaging modality to provide details on vascular complication. Both were carried out in rest and provocative positions (Figure 3). Venograms were carried out in 9 (75%) of the VTOS cases. Table 1 provides further details and imaging findings.

**Table 1:** - Investigations and findings.

Imaging	n (%)
***Plain thoracic inlet and cervical spine x-ray***	90 (100)
Cervical rib	76 (84.4)
Anomalous 1^st^ rib	12 (12.1)
Prominent 7^th^ cervical transverse process	2 (2.2)
***Duplex scan (rest and provocation)***	90 (100)
Post-stenotic dilatation	9 (10)
Subclavian artery aneurysm	10 (11.1)
Arterial thrombosis	7 (7.8)
Computed tomography angiography (rest and provocation)	56 (62.2)
Conventional angiogram (rest and provocation)	27 (30.0)
Venogram	9 (10.0)

Values are presented as number and precentage (%).

We followed the supraclavicular technique in all cases. All patients underwent decompression, scalenectomy, and histopathological examination which revealed hypertrophic features. Total cervical rib resection was carried out in 75 (83.3%) cases. First rib was resected in 33 (36.7%) cases. Total vascular surgeries were carried out in 20 (22.2%) cases. An endovascular approach using catheter-directed thrombolysis and subclavian vein stent placement was carried out in 2 (16.7%) VTOS cases (Table 2).

**Table 2:** - Operative details.

Extent of resection	n (%)
*Decompression surgery*	
Total cervical rib alone	54 (60.0)
Cervical rib and first rib	21 (23.3)
First rib and fibrous band resection	12 (13.3)
Partial cervical rib and fibrous band	2 (2.2)
Elongated C7 transverse process and fibrous band	2 (2.2)
***Vascular procedure***	
Arterial repair	18 (20.0)
***Repair of subclavian artery aneurysms***	10 (11.1)
Resection and interposition graft repair	7 (5.6)
Venous (GSV) graft	4 (4.4)
Synthetic (PTFE) grafts	3 (3.3)
Resection and end to end anastomosis	3 (3.3)
***Revascularization surgery***	8 (8.9)
Axillary-brachial bypass (using vein)	1 (1.1)
Axillary-distal thrombectomy	7 (7.8)
Venous repair	2 (2.2)
***Endovenous intervention***	
Catheter directed thrombolysis and stent of subclavian vein	2 (2.2)

Values are presented as a number and precentage (%). GSV: great saphenous vein, PTFE: polytetrafluoroethylene

There were no cases of post-operative mortality or recurrence. The overall post-operative complication rate was 18.9%. Wound hematoma was observed in 2 (2.2%) cases and was managed conservatively. Post-operative sensorimotor deficits were observed in 9 (10%) cases. Sensorimotor symptoms consisted mainly of minor weakness at the finger or wrist level or numbness over the distribution of the brachial plexus nerves. All patients underwent physiotherapy and improved within 1-3 months. A total of 4 (4.4%) cases were complicated by pneumothorax, all of which were managed with a chest tube, and recovered within 24-48 hours. Two (2.2%) patients had chylothorax, and one (1.1%) of them also had supraclavicular lymphocele which were managed with a chest tube, drainage, fat-free diet, and total parenteral nutrition and recovered within one month. All patients had their recovery measured using Derkash’s classification, and all patients categorized their recovery as “excellent” immediately or at the end of the follow-up period (Table 3).

**Table 3:** - Post-operative complication outcomes after 1, 6, and 12 months.

Postoperative complications	n (%)	Presence of complications		
		1 month	6 months	12 months
Wound hematoma	2 (2.2)	0 (0.0)	0 (0.0)	0 (0.0)
Sensorimotor symptoms	9 (10.0)	8 (8.9)	0 (0.0)	0 (0.0)
Pneumothorax	4 (4.4)	0 (0.0)	0 (0.0)	0 (0.0)
Chylothorax	2 (2.2)	0 (0.0)	0 (0.0)	0 (0.0)
Supraclavicular lymphocele	1 (2.2)	0 (0.0)	0 (0.0)	0 (0.0)

Values are presented as a number and precentage (%).

## Discussion

The term TOS was first coined by Peet et al^7^ in 1956, when they described a group of neurovascular symptoms caused by compression of the neurovascular structures from the neck base to the arm via the axilla. Since then, multiple centers have reported their experiences particularly with NTOS due to its relatively common prevalence.^5,8^ From 1980-2015, a systematic review by Peek et al^5^ included only 7 studies that reported operative interventions and outcomes for vascular TOS, with a total of 159 cases most of which were VTOS. The low reported number in their study was credited to the lack of universal reporting standards.^5^ Furthermore, the availability of large series was limited by the fact that only 5% of TOS were VTOS and 1% were ATOS.^2^ Moreover, in the absence of ischemic events, ATOS may be underestimated, as it can be masked by neurogenic compression symptoms.^9^ Worldwide, many centers are underutilizing non-invasive vascular imagings such as duplex scans, which may also lead to underestimate vascular TOS.^10^ In contrast to published data, the majority (86.7%) of cases in this study were ATOS.^2^

Furthermore, this study was carried out in a tertiary referral hospital; therefore, the study settings, such as under-reference, may have affected the prevalence. In addition, patients with VTOS are usually young athletes, presenting with signs and symptoms of DVT that are treated with anticoagulation therapy, forgoing further investigation for secondary mechanical DVT causes.^11^

All the cases included in this study were symptomatic. In addition to clinical diagnosis, other investigation methods were used for confirmation. Plain radiographs were successful in identifying the compressing bony structures in this study, similar to a study by Ciampi et al^12^ showing that the majority of compressing bones were cervical ribs (84.4%). Duplex scan is a non-invasive, simple, cheap, and easy clinical test. Furthermore, it can identify vascular hemodynamic and structural changes. In symptomatic TOS patients, duplex scan was successful in identifying vascular TOS cases in this study. A cut-off of blood flow with reproduction of symptoms during provocative maneuver (arm hyperabduction) was most suggestive of ATOS. Duplex scans were successful in identifying post-stenotic subclavian artery aneurysms in 8 cases and axillary or distal arterial thrombosis in 7 cases. All duplex examinations were carried out in an upright position, which has superior sensitivity and eliminated the need for MRI.^13^ Surgical outcomes are better in patients who undergo duplex examination, as it provides clear evidence of arterial compression.^5^ However, as duplex scans enable assessment of blood flow based on indirect signs of the proximal artery, adequate exposure of the subclavian artery is obscured by the clavicle.^10^ Therefore, the use of other imaging modalities such as CTA and MRI with the thoracic outlet protocol can yield more anatomical details.^14^ Computed tomography arterio/venography was used in 62.2% of cases, and its availability and speed were the major reasons for choosing this method over MRI. Moreover, CTA showed superior accuracy in detecting ATOS.^9^ Computed tomography angiography aided in identifying the site and cause of vascular damage along with the presence of associated emboli, as well as providing a detailed relationship of bony deformity or fibrous bands with vascular compression, without the need for invasive conventional angiography in most cases. Computed tomography angiography also leads to favorable surgical outcomes and better selection of operative candidates.^14,15^ Ionizing radiation, iodinated contrast, and less soft tissue details are all limitations of CTA. Magnetic resonance imaging is a better option in patients who are suspected of having NTOS, when a compressing soft tissue structure is suspected, or when CTA is contraindicated.^12^ Conventional angiography was used in 30% of cases, mainly before 2006, and was later reserved for complex cases or when an endovascular approach was planned. It provides an excellent assessment of digital, hand, and forearm arteries, and allows for a dynamic view of the subclavian and axillary arteries. However, its use has become limited owing to its invasiveness and limitations in cases of aneurysms with intraluminal thrombi.^16^

Vascular TOS requires surgical decompression.^5^ Different decompression approaches were described: transaxillary, infraclavicular, or supraclavicular. The majority of published studies have used the transaxillary approach.^5^ Here, we report our experience with the supraclavicular approach by which all patients reported “excellent” recovery. The supraclavicular approach provides better vascular exposure and has been associated with excellent outcomes in multiple studies.^12,17^ Furthermore, it allows surgeons to carry out intraoperative provocative maneuvers in order to identify the site of compression and determine the efficacy of release.

However, this approach provides poor cosmetic outcomes. Vascular reconstruction is usually achieved by resection and anastomosis, as well as replacement with a vein or prosthetic graft.^5^ Grafts were used in 5 cases (3 veins and 2 PTFE grafts), and during the follow-up period, no graft complications were encountered. In a separate ATOS study, PTFE bypass occlusion was identified in the 4^th^ month post-surgery in one out of 7 cases that did not undergo decompression surgery.^18^ The PTFE graft was replaced with a vein graft, which remained patent for the 12-year duration of the study.^18^ With regard to ATOS, there is limited data regarding patency rates, need for reoperation, superiority of vein versus PTFE grafts, and rarity.^5^ In the VTOS group, we had only 2 patients who presented with acute DVT and underwent catheter-directed thrombolytic therapy followed by surgical decompression and a period of anticoagulation, and then received subclavian vein stenting due to residual stenosis. Catheter-directed thrombolytic therapy shows superiority to anticoagulation for subclavian vein thrombosis due to TOS.^19^ We did not encounter any recurrence in this series.

### Study limitations

The retrospective nature of the study design and the short-term follow-up period were this study limitations. Patients with satisfactory outcomes are usually reluctant to undergo another visit and examination; therefore, they often fail to return for follow-up visits. Furthermore, minor complications were reported, comparable to those reported for transaxillary and supraclavicular approaches.^12,18^

Endovascular treatment of vascular TOS usually requires decompression surgery to avoid stents fracture. Furthermore, long-term stent patency rates remain unclear.^20,21^ Many interventionists treat vascular TOS as if it was an atherosclerotic lesion; once they observe a stenosis via angiography, a stent is inserted without subsequent decompression surgeries leading to stent occlusion. This approach makes correction surgeries difficult.^21^ Thoracic outlet syndrome is a growing topic in the field of upper limb ischemia, and the unfamiliarity of both patients and physicians with this clinical entity provides more importance to providing a gamut of credible experiences on this topic. We base our excellent results on appropriate patient selection, adequate diagnosis, operative technique, and performance of these operations by vascular surgeons. With an equivalently complex case, vascular surgeons had fewer complications than other surgeons, and better outcomes owing to their microvascular and endovascular skills.^22^

In conclusion, vascular TOS can affect productivity of society, and in particular, the young working population. Clinical presentation coupled with plain thoracic inlet radiography and duplex scanning gives desirable details as an initial step. Computed tomography angiography can be used as a second-line imaging technique. Other imaging modalities were selected on a case-by-case basis. Surgery is the standard of care for patients with vascular TOS. In our experience, the supraclavicular approach provides desirable vascular outcomes. The risk of pneumothorax, thoracic duct injury, and poor aesthetics remain drawbacks of this approach. Multicenter large-volume studies concerning vascular TOS diagnosis and management are required.
